# Post-Pandemic Genomic Diversity and Lineage Turnover of Influenza Viruses in Mexico During 2022–2023

**DOI:** 10.3390/v18060609

**Published:** 2026-05-27

**Authors:** Blanca Taboada, Selene Zárate, José Esteban Muñoz-Medina, Joel Armando Vazquez-Perez, Alejandro Sanchez-Flores, Angel Gustavo Salas-Lais, Alejandro Uscanga Junco, Alida Zárate, Larissa Fernandes-Matano, Luis Antonio Uribe-Noguez, Enrique Mendoza-Ramírez, Fidencio Mejía-Nepomuceno, Carlos F. Arias

**Affiliations:** 1 Departamento de Genética del Desarrollo y Fisiología Molecular, Instituto de Biotecnología, Universidad Nacional Autónoma de México, Cuernavaca 62210, Morelos, Mexico; alida.zarate@ibt.unam.mx (A.Z.); carlos.arias@ibt.unam.mx (C.F.A.); 2 Posgrado en Ciencias Genómicas, Universidad Autónoma de la Ciudad de México, Ciudad de México 03100, Mexico; 3 Coordinación de Calidad de Insumos y Laboratorios Especializados, Instituto Mexicano del Seguro Social, Ciudad de México 07760, Mexico; eban10@hotmail.com (J.E.M.-M.); salas_lais@yahoo.com.mx (A.G.S.-L.); larissamatano@gmail.com (L.F.-M.); 4 Instituto Nacional de Enfermedades Respiratorias Ismael Cosío Villegas, Ciudad de México 14080, Mexico; joel.vazquez@iner.gob.mx (J.A.V.-P.); heinrichunam@gmail.com (E.M.-R.); biolfimene@gmail.com (F.M.-N.); 5 Unidad Universitaria de Secuenciación Masiva y Bioinformática, Instituto de Biotecnología, Universidad Nacional Autónoma de México, Cuernavaca 62210, Morelos, Mexico; alejandro.sanchez@ibt.unam.mx; 6 Instituto de Investigación en Ciencias Básicas Aplicadas (IICBA), Universidad Autónoma del Estado de Morelos, Cuernavaca 62210, Morelos, Mexico; usju5296@gmail.com; 7 Laboratorio Central de Epidemiología, Instituto Mexicano del Seguro Social, Ciudad de México 02990, Mexico; luis.uribe@imss.gob.mx

**Keywords:** influenza, Mexico, virus evolution, genomic surveillance

## Abstract

Seasonal influenza circulation was profoundly disrupted during the COVID-19 pandemic, resulting in an unprecedented global decline in viral activity and genetic diversity. As non-pharmaceutical interventions gradually relaxed, influenza viruses re-emerged in multiple regions in 2022; however, genomic data from Latin America remain limited, particularly for the first post-pandemic season. In this study, we analyzed the epidemiological, genomic, and evolutionary patterns of influenza A and B viruses circulating in Mexico between June 2018 and June 2023, with a specific focus on the first post-pandemic season (June 2022–June 2023). More than 90% of the influenza virus genomes available from Mexico for this post-pandemic period were generated in this study, while data from earlier seasons were used for contextualization. Following a near-complete absence of influenza circulation during 2020–2021, viral activity re-emerged in late 2021 and intensified during 2022–2023. Post-pandemic circulation in Mexico was dominated by influenza A(H3N2), with a lower contribution from A(H1N1)pdm09 and a delayed re-emergence of B/Victoria; B/Yamagata was not detected. Genomic analyses revealed rapid lineage turnover after the pandemic, characterized by the predominance of post-pandemic clades and reduced genetic diversity compared with pre-pandemic seasons. Phylogenetic analyses indicated multiple introductions and strong regional connectivity across North and South America.

## 1. Introduction

Seasonal influenza continues to be a significant contributor to global respiratory illness, resulting in a billion infections and between 300,000 and 600,000 respiratory deaths annually. International surveillance of the disease is coordinated by the World Health Organization (WHO) through the Global Influenza Surveillance and Response System (GISRS) and supported by the Global Influenza Hospital Surveillance Network (GIHSN). The information generated by this effort is essential for monitoring viral circulation, tracking genetic evolution, and guiding seasonal vaccine strain selection [1,2,3,4,5,6,7,8,9,10]. Long-term genomic surveillance studies demonstrated that influenza virus evolution is characterized by high genetic diversification, recurrent lineage replacement, and regional persistence of some lineages [5,6,7,8,9,11,12,13,14,15,16,17]. It has been widely reported that the COVID-19 pandemic significantly altered the circulation patterns of many respiratory pathogens [10,17]. In the case of influenza, a sharp decline in cases was observed from March 2020 to most of 2021, probably due to the implementation of non-pharmaceutical interventions, including mask use, school closures, and travel restrictions [10,17].

As these interventions were gradually relaxed, influenza activity re-emerged in many regions during 2022. Genomic surveillance data from Australia and some Asian countries (Australia, Turkey, Myanmar, Bhutan, Pakistan, and China) documented rapid lineage replacement and the emergence of newly drifted clades of A(H3N2), A(H1N1)pdm09, and B/Victoria viruses, indicating a rapid re-establishment of influenza evolutionary dynamics following the pandemic period [9,16,18,19]. In contrast, genomic data from Latin America remains limited, particularly for the first post-pandemic influenza season in 2022–2023. This lack of data restricts regional analyses of post-pandemic lineage turnover and the relative contributions of imported versus locally circulating strains [20].

The reactivation of influenza genomic surveillance in Mexico in mid-2022 provides a unique opportunity to assess the re-establishment of influenza virus diversity following the COVID-19 pandemic. This study examines the genomic diversity, temporal dynamics, and phylogenetic relationships of influenza A and B viruses circulating in Mexico from June 2018 to June 2023, with a particular focus on viruses sequenced during the first post-pandemic season (June 2022 to June 2023). More than 90% of the influenza virus genomes from Mexico for this period were generated as part of this study. In addition to whole-genome influenza sequencing, to determine clustering of Mexican sequences, phylogenetic analyses based on the hemagglutinin (HA) and neuraminidase (NA) genes were performed. Finally, mutational analyses were conducted across all viral segments to characterize and compare Mexican influenza viruses with those circulating elsewhere. Genomic and epidemiological data were integrated to provide a comprehensive overview of post-pandemic influenza evolution in Mexico.

## 2. Materials and Methods

### 2.1. Epidemiological Data

Weekly counts of influenza cases were obtained from the FluNet database [20,21], the global influenza surveillance system maintained by the WHO through the Division of Emerging and Other Communicable Diseases Surveillance and Control. FluNet compiles laboratory-confirmed influenza surveillance data reported by participating countries and regions worldwide.

For this study, epidemiological data from 2018 to 2023 were analyzed for Mexico and for two comparative regional groups: Temperate South America, including Argentina, Chile, Paraguay, and Uruguay; and North America, including Bermuda, Canada, and the United States of America. These data were used to characterize temporal trends in influenza activity and to contextualize the epidemiological patterns observed in Mexico.

### 2.2. Sample Collection and Sequencing

Respiratory samples from patients testing positive for influenza A or B viruses were collected through the national network of public health laboratories for the national surveillance of respiratory viruses, which includes the Instituto Mexicano del Seguro Social (IMSS) and the Instituto Nacional de Enfermedades Respiratorias (INER). All procedures (RNA extraction, cold storage, and transport to the reference laboratory) were performed according to standardized protocols validated by the Instituto de Diagnóstico y Referencia Epidemiológicos (InDRE), Secretaría de Salud, Mexico, and were approved by the WHO [22].

For genomic analyses, the selected samples were based on the monthly national epidemiological trends and the states reporting the highest number of cases between June 2022 and June 2023. RNA samples were processed following the protocol described by Lin et al. [23]. Influenza A viruses were amplified using primers reported by Zhou and Wentworth [24], while influenza B viruses were amplified using primers described by Zhou et al. [25]. Sequencing libraries were prepared using the Illumina^®^ COVIDSeq™ Assay kit (San Diego, CA, USA), and sequencing was performed on an Illumina NextSeq 500 platform using a 2 × 150 bp paired-end configuration. In total, 1040 influenza-positive samples from 19 Mexican states were successfully sequenced.

### 2.3. Genome Assembly and Consensus Generation

Reference sequences corresponding to the WHO-recommended vaccine strains for the 2022–2023 Northern Hemisphere influenza season were retrieved from the Global Initiative on Sharing All Influenza Data (GISAID) and used as reference genomes for each influenza subtype or lineage: A/Victoria/2570/2019 for H1N1pdm09 (EPI_ISL_417210), A/Darwin/9/2021 for H3N2 (EPI_ISL_2233240), B/Austria/1359417/2021 for the B/Victoria lineage (EPI_ISL_1519459), and B/Phuket/3073/2013 for the B/Yamagata lineage (EPI_ISL_168822) as the historical reference for the B/Yamagata lineage, which was included for completeness despite the absence of post-pandemic circulation.

Raw paired-end sequencing reads were processed using a custom bioinformatics pipeline developed for influenza genome reconstruction. First, reads were preprocessed using fastp v0.23.4 to remove adapter sequences and low-quality bases [26]. Then, quality-filtered reads were mapped against the indexed reference genomes using Bowtie2 v2.5.0 in --very-sensitive-local mode [27]. For each sample, the best-matching reference genome was selected based on alignment depth across the hemagglutinin (HA) and neuraminidase (NA) segments. SAM files were converted to BAM format, sorted, and indexed using Samtools v1.10 [28]. Consensus genomes were generated using iVar v1.3.1 [29], with a minimum coverage depth of ≥20× and a base quality threshold of Q20. Positions below this threshold were masked as ambiguous bases (“N”). No intrahost single-nucleotide variant or minor-variant analyses were performed.

The complete bioinformatics pipeline is available at: https://github.com/BlancaTaboada/Influenza_genomes_pipeline (accessed on 10 January 2026). All influenza genome sequences generated in this study were submitted to the GISAID and GenBank databases, and the corresponding accession numbers are provided in Table S1. To evaluate whether the monthly sequencing output reflected the temporal distribution of influenza activity during the study period, Spearman’s rank correlation coefficient was calculated between the monthly number of influenza samples with at least one high-quality consensus segment generated in this study and the monthly number of laboratory-confirmed influenza cases reported in Mexico between June 2022 and June 2023.

### 2.4. Genomic and Phylogenetic Analyses

Clade and subclade assignment were performed using Nextclade (https://clades.nextstrain.org/, accessed on 15 January 2026), using the same vaccine strain reference sequences for influenza A/H3N2, A/H1N1pdm09, and influenza B (Victoria lineage) described in the previous section. For genetic diversity and temporal frequency analyses, all available influenza virus sequences from Mexico collected between 2018 and 2023 were retrieved from the GISAID EpiFlu database and analyzed together with the genomes generated in this study. This public dataset included 461 sequences collected before the post-pandemic study period, comprising 242 A/H1N1pdm09, 139 A/H3N2, and 80 influenza B sequences, as well as 75 sequences collected during the same post-pandemic period analyzed here, including 8 A/H1N1pdm09, 52 A/H3N2, and 15 influenza B sequences. These sequences can be accessed in the EpiFlu database using the number EPI_SET_260319uq.

To reduce sampling noise in monthly clade-frequency estimates, only months with at least 17 Mexican sequences, corresponding to the median monthly sample size, were included. Influenza B/Yamagata was evaluated but excluded from downstream analyses due to the absence of post-pandemic circulation. To assess potential geographic sampling bias, we compared the regional distribution of samples with at least one high-quality consensus segment with the regional distribution of laboratory-confirmed influenza cases reported in Mexico during the same period. This comparison was performed at the regional level, using the six-region framework applied throughout the manuscript: Northwest (NW), Northeast (NE), West (W), Central–North (CN), Central–South (CS), and Southeast (SE) (Supplementary Figure S1). For each region, we calculated the proportion of genomes and confirmed cases and used the difference between these proportions to identify over- and under-represented regions.

For phylogenetic analyses, representative influenza virus sequences collected between 2014 and 2023 were retrieved from the GISAID EpiFlu database, along with the corresponding vaccine strain reference sequence for each influenza type. To ensure balanced temporal and geographic representation, an equal number of sequences per continent was sampled for each month and year, with a higher proportion of sequences sampled from recent years compared to earlier periods. These sequences can be retrieved from EpiFlu using the accession number EPI_SET_260204tx. In addition, all Mexican sequences were included, with a maximum of 20 sequences per month/year. Only viruses with full-length sequences of segments 4 (HA) and 6 (NA) were retained for analysis.

In total, 1652 A/H3N2, 1495 A/H1N1pdm09, and 1308 influenza B sequences were included in the phylogenetic analyses. Hemagglutinin (HA) and neuraminidase (NA) sequences for each influenza type were aligned separately using MAFFT v7 [30]. Maximum likelihood phylogenetic trees were inferred using IQ-TREE2 [31], applying the best-fitting nucleotide substitution models identified by ModelFinder: TVM + F + I + R3 for H3, N2, and B/NA; GTR + F + I + R4 for H1; GTR + F + I + G4 for N1; and TVM + F + I + R2 for B/HA. All phylogenies were time-scaled using sample collection dates with LSD2. Trees were visualized and annotated in R using the ggtree package [32].

Potential reassortment events among influenza viruses generated in this study were screened using K-FluDB, a k-mer-based database designed for influenza A genomic surveillance [33]. Briefly, sequencing reads from influenza A-positive samples were mapped against subtype-specific k-mers defined in K-FluDB. Subtype assignments were evaluated at the segment level, and samples showing discordant subtype-specific signals between HA/NA and internal genomic segments were considered potential reassortants.

Amino acid sequences derived from all viral segments, including HA, NA, PB2, PB1, PB1-F2, PA, NP, M1, M2, NS1, and NS2, were analyzed using FluSurver (https://flusurver.bii.a-star.edu.sg/, accessed on 15 January 2026) to identify amino acid substitutions relative to the egg-propagated vaccine strains described in Section 2.3. This analysis was restricted to the post-pandemic Mexican dataset described above, comprising the genomes generated in this study and the 75 additional Mexican genomes collected during the same period and retrieved from GISAID. For downstream interpretation, we retained only recurrent mutations detected in at least 5% of sequences for each protein and subtype that were assigned an interest level of 2 or 3 by FluSurver, corresponding to substitutions with known or predicted biological relevance. These included markers associated with antiviral drug susceptibility, virulence, host-cell specificity, and antigenic change. N-linked glycosylation motifs (N-X-S/T, where X can be any amino acid except P) were identified separately using Nextclade (https://clades.nextstrain.org/, accessed on 15 January 2026).

## 3. Results

### 3.1. Seasonal Influenza Epidemiology Before and After the COVID-19 Pandemic

**Figure 1:**
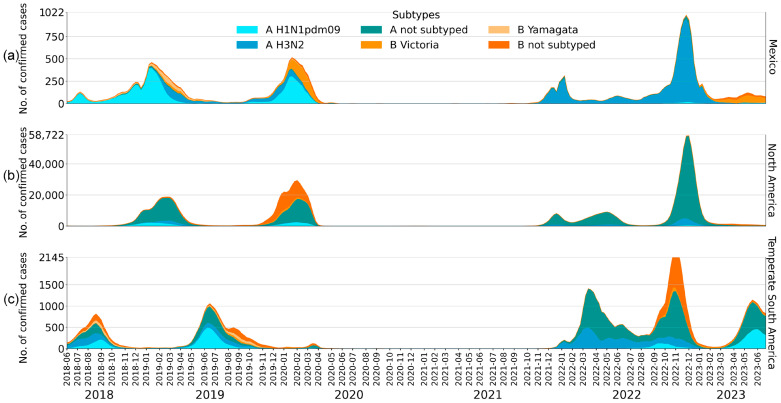
Seasonal circulation of influenza virus subtypes before and after the COVID-19 pandemic (June 2018–June 2023). Stacked area plots show the weekly number of confirmed influenza cases by subtype. (**a**) Mexico. (**b**). North America. (**c**) Temperate South America.

Using public data from the WHO’s database, Flunet, the circulation of different influenza subtypes was analyzed. As can be observed in Figure 1a, during the two seasons prior to the COVID-19 pandemic, influenza activity in Mexico was characterized by winter annual peaks, with epidemic waves dominated by Influenza A viruses. Most of the cases were due to the A(H1N1)pdm09 subtype, with a smaller but consistent contribution from A(H3N2) and B(Victoria) (Figure 1a). On the other hand, B/Yamagata was detected only sporadically.

As shown in Figure 1a, this seasonal pattern was disrupted by the COVID-19 pandemic in early 2020, when influenza circulation declined across all subtypes. This interruption was observed not only in Mexico, but also in North (Figure 1b) and South America (Figure 1c), as well as globally [3,34], indicating a large-scale phenomenon rather than a region-specific effect.

Influenza circulation re-emerged in late 2021 and throughout 2022, although with fewer cases than in pre-pandemic seasons. During the 2021–2022 winter season, circulation in Mexico was mainly driven by A(H3N2), whereas A(H1N1)pdm09 remained at relatively low levels, and influenza B/Victoria accounted for a smaller proportion of cases. In North America, however, much of the post-pandemic Influenza A activity was reported as not subtyped, limiting direct comparison with the subtype distribution observed in Mexico. In South America, influenza activity during this season was driven by both A(H3N2) and unsubtyped Influenza A viruses.

Notably, before the major 2022–2023 winter epidemic peak, an earlier off-season increase in influenza activity was observed during atypical months. In Mexico and North America, this was evident as a smaller rise in cases around May–June 2022, whereas in South America influenza activity remained more extended across several months of 2022.

During the 2022–2023 winter season, all three regions showed a marked epidemic peak. In Mexico, this season was dominated by A(H3N2) and represented the largest influenza peak since 2019. North America exhibited a broadly similar temporal pattern, although a substantial fraction of Influenza A cases remained unsubtyped. In South America, the peak involved co-circulation of A(H3N2) and B/Victoria viruses. As previously reported, B/Yamagata remained undetected across regions during the post-pandemic period [35].

### 3.2. Sequencing Efficiency

**Figure 2:**
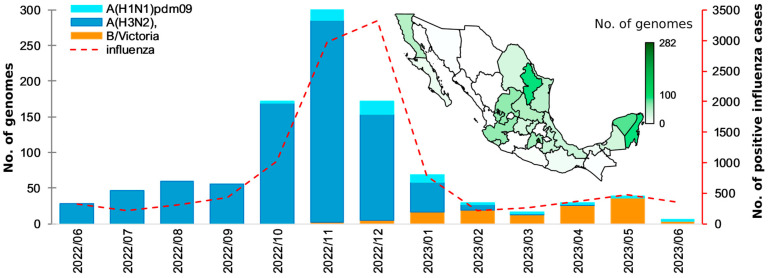
Monthly genomic surveillance of influenza viruses in Mexico during the first post-pandemic season (June 2022–June 2023). The dashed red line indicates the monthly number of laboratory-confirmed influenza cases (right *y*-axis). The inset map depicts the geographic distribution of sequenced genomes across Mexico, with color intensity representing the number of genomes obtained per state during the study period.

To characterize the circulation of influenza viruses in Mexico during the first post-pandemic influenza season (Figure 2), the whole genome of 1040 influenza-positive samples from 19 Mexican states, collected between June 2022 and June 2023, were sequenced. Of these, 905 samples yielded at least one high-quality influenza consensus segment and were submitted to the GISAID and GenBank databases (Table S1), including 792 Influenza A and 113 Influenza B samples. Overall, 6141 genomic segments were recovered across these samples, with subtype-specific recovery summarized in Table S1.

Sequencing and assembly efficiency varied among viral segments and subtypes (Table S2). The highest complete-segment recovery was obtained for NS (*n* = 898), followed by MP (*n* = 854), NA (*n* = 852), and HA (*n* = 841). In contrast, the lowest recovery was observed for PB1 (*n* = 468), particularly among A(H1N1)pdm09 samples. Notably, HA and NA, the two surface glycoprotein segments used in subsequent analyses, were recovered from most samples. Complete genomes were obtained for 49.9% samples, while the remaining samples produced between one and seven high-quality segments (Table S2).

The number of influenza genomes per month matched with the temporal distribution of confirmed influenza cases, as shown in Figure 2. Furthermore, a strong positive correlation was observed between the monthly number of genomes and the confirmed cases (Spearman’s ρ = 0.82), indicating that sequencing output was representative of the epidemiological curve during the study period. Most of the samples were from Mexico City (*n* = 184), followed by Nuevo León (*n* = 81), Quintana Roo (*n* = 87), Yucatán (*n* = 70), and Zacatecas (*n* = 53) (Table S3).

To assess whether geographic sampling reflected regional influenza activity, we compared the proportion of sequenced genomes with the proportion of laboratory-confirmed cases by region (Supplementary Table S3). Genomic sampling broadly captured regional influenza activity, although some imbalances were observed. W, NE, and CS were relatively proportional to their reported case burden, whereas SE was over-represented (24.9% of genomes vs. 10.6% of cases) and CN was under-represented (16.0% of genomes vs. 26.7% of cases). Thus, the dataset should be interpreted as a genomic surveillance dataset rather than as a fully population-representative sample of influenza circulation across Mexico.

### 3.3. Genetic Diversity and Phylogenetic Analysis of Influenza

To investigate the genetic diversity and temporal dynamics of influenza viruses in Mexico, we analyzed the 2018–2023 genomic dataset described in the Methods section, retaining only months with at least 17 genomes to reduce sampling noise. After applying this threshold, the pre-pandemic dataset comprised 423 genomes, including 235 (55.6%) A/H1N1pdm09, 109 (25.8%) A/H3N2, and 79 (18.7%) influenza B genomes, of which 41 corresponded to B/Yamagata and 38 to B/Victoria. In contrast, the post-pandemic dataset comprised 980 genomes, including 797 (81.3%) A/H3N2, 62 (6.3%) A/H1N1pdm09, and 121 (12.4%) B/Victoria genomes. This dataset allowed us to compare historical pre-pandemic diversity with the viral composition observed during the first post-pandemic influenza season in Mexico.

#### 3.3.1. Influenza A(H1N1)pdm09

**Figure 3:**
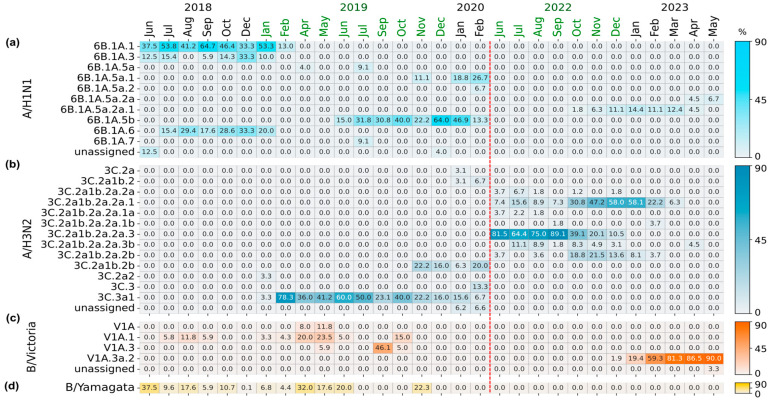
Temporal dynamics of influenza virus HA clades circulating in Mexico (2018–2023). Monthly relative frequencies of HA clades for (**a**) influenza A(H1N1)pdm09, (**b**) influenza A(H3N2), (**c**) influenza B/Victoria, and (**d**) influenza B/Yamagata. Only months with at least 17 sequences were included to ensure reliable estimation of relative clade frequencies. The dashed vertical line marks the onset of the COVID-19 pandemic-associated interruption of influenza circulation.

The genetic diversity of influenza A(H1N1)pdm09 viruses circulating in Mexico showed temporal structuring before and after the COVID-19 pandemic (Figure 3a). During the pre-pandemic period (2018–2020), multiple HA clades derived from 6B.1A co-circulated, with 6B.1A.1 and 6B.1A.6 subclades predominating in 2018, alongside NA clade A.1.1 (Figure S2a). By mid-2019, these lineages were progressively replaced by 6B.1A.5b, accompanied by the emergence of NA clade B.1, followed in early 2020 by 6B.1A.5a.1 and NA clade B.2.1, reflecting continuous lineage turnover prior to the pandemic interruption.

After a halt in positive samples during 2020–2022, A(H1N1)pdm09 re-emerged in late 2022 with a reduced but genetically structured population, particularly subclade 6B.1A.5a.2a.1 (Figure 3a). In parallel, NA clades C.5.1.1, C.5.2, and C.5.3 replaced pre-pandemic A- and B-class clades and dominated circulation through mid-2023 (Figure S2a).

**Figure 4:**
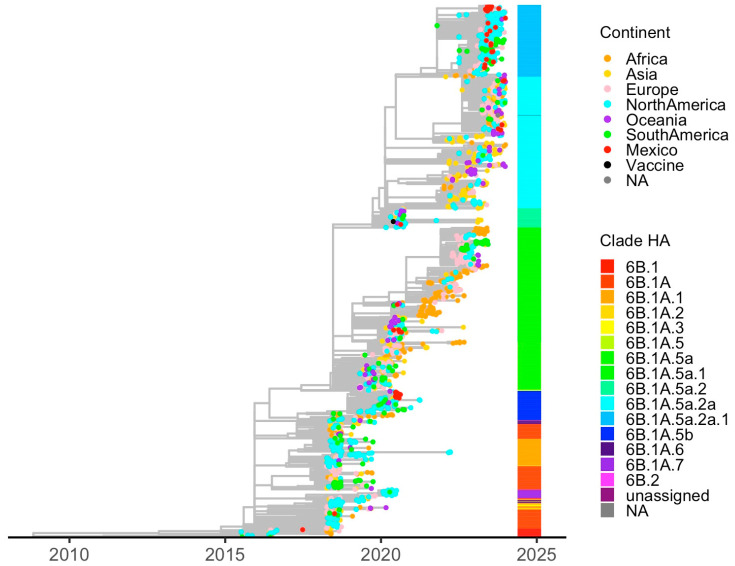
Maximum likelihood phylogenetic tree for HA Influenza A subtype H1. The tips of the tree are colored by sampling location, and the heatmap alongside the tips shows the HA clade assignment for each sequence. The vaccine strain contemporary to the Mexican sequences reported in this study is marked in black.

Phylogenetic reconstruction of HA sequences (Figure 4) showed that recent Mexican A(H1N1)pdm09 viruses clustered tightly within 6B.1A.5a.2a and 6B.1A.5a.2a.1, consistent with their global expansion since 2021. Older Mexican sequences grouped within 6B.1A.5b, which circulated globally during 2019–2020. NA phylogenies (Figure S3) mirrored these patterns, with post-pandemic Mexican viruses clustering within C-class clades. In general, HA and NA Mexican sequences showed close genetic relationships with sequences from North America, indicating regional connectivity.

#### 3.3.2. Influenza A(H3N2)

Influenza A(H3N2) exhibited greater genetic diversity than A(H1N1)pdm09, particularly following its post-pandemic re-emergence (Figure 3b). No A(H3N2) detections were recorded in Mexico in 2018. During 2019, circulation was dominated by HA clade 3C.3a1, accompanied by NA clade A.2.1 (Figure S2b). By late 2019 and early 2020, these viruses were progressively replaced by 3C.2a1b.2b, reflecting global trends prior to the pandemic.

A(H3N2) re-emerged in mid-2022 with pronounced genetic diversification. HA clades derived from 3C.2a1b.2a dominated circulation, particularly 3C.2a1b.2a.2a.1 and 3C.2a1b.2a.2a.3, which defined the 2022–2023 influenza season in Mexico. Additional subclades, including 3C.2a1b.2a.2a.3b and 3C.2a1b.2b, circulated at lower frequencies, indicating ongoing diversification. NA evolution followed a similar replacement pattern. NA clade B.3 predominated during early 2022, followed by a transition toward B.2, which became dominant by late 2022 and persisted into 2023.

**Figure 5:**
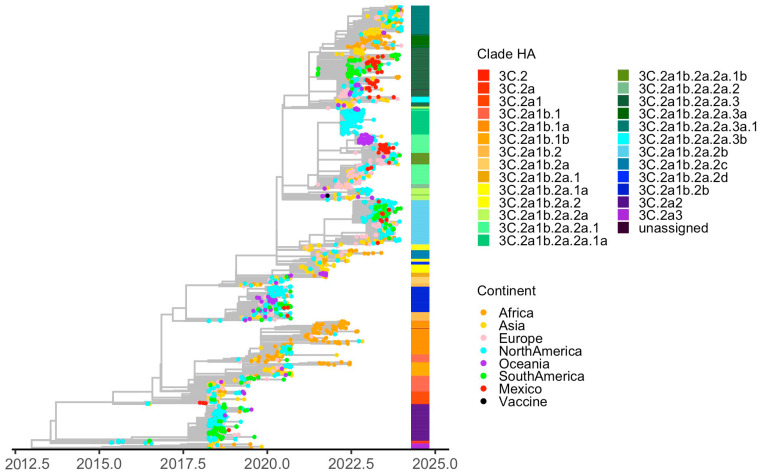
Maximum likelihood phylogenetic tree for HA Influenza A subtype H3. The tips of the tree are colored by sampling location, and the heatmap alongside the tips shows the HA clade assignment for each sequence. The vaccine strain contemporary to the Mexican sequences reported in this study is marked in black.

Phylogenetic analyses of HA sequences (Figure 5) revealed that Mexican A(H3N2) viruses were distributed across multiple post-pandemic clades and clustered closely with sequences from both North and South America, consistent with repeated introductions and regional mixing. NA phylogenies (Figure S4) further supported this interpretation, with Mexican sequences forming distinct clusters within B.2 and B.3, some of which were enriched for sequences from specific continental origins.

#### 3.3.3. Influenza B/Victoria Lineage

Before the COVID-19 pandemic, B/Victoria viruses circulated at moderate levels in Mexico with limited genetic diversity. HA clade V1A.1, and NA clade A.1, predominated between 2018 and mid-2019, with sporadic detection of V1A.3 toward late 2019 (Figure 3c).

Following a two-year absence, B/Victoria re-emerged in early 2023, marking a delayed recovery compared with influenza A subtypes. Post-pandemic viruses were almost exclusively assigned to V1A.3a.2, which rapidly replaced pre-pandemic lineages. NA sequences clustered predominantly within B, B.7 and B.7.1 (Figure S2c), consistent with the predominance and genetic diversification lineages circulating globally during the 2021–2023 influenza season [36,37].

**Figure 6:**
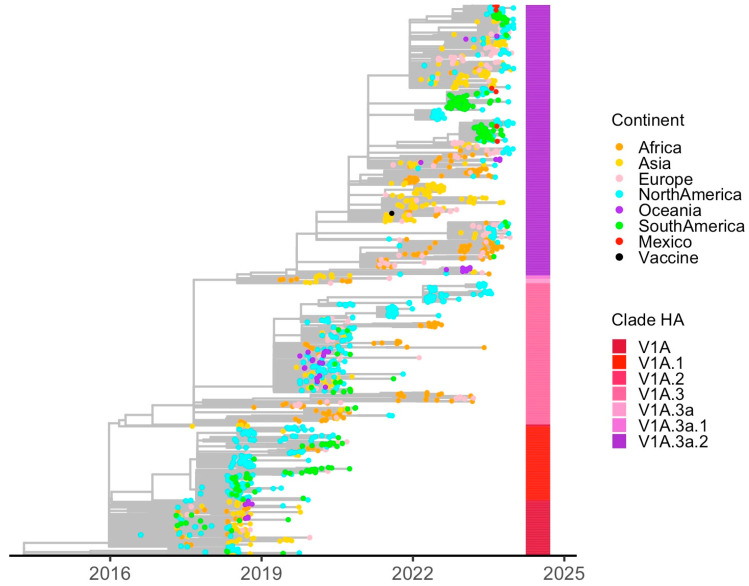
Maximum likelihood phylogenetic tree for HA Influenza B subtype Victoria. The tips of the tree are colored by sampling location, and the heatmap alongside the tips shows the HA clade assignment for each sequence. The vaccine strain contemporary to the Mexican sequences reported in this study is marked in black.

Phylogenetic analyses of HA (Figure 6) showed that Mexican B/Victoria viruses formed several clusters within the V1A.3a.2 clade, composed almost exclusively of sequences from the Americas, suggesting regional circulation following reintroduction. NA phylogenies (Figure S5) supported this pattern. Despite lower overall activity compared with influenza A viruses, the dominance of a single HA lineage indicates a strong post-pandemic bottleneck followed by rapid lineage expansion. This evolutionary pattern is consistent with reports from other Northern Hemisphere countries, reflecting the synchronized re-establishment of the B/Victoria genetic pool during the 2022–2023 influenza season.

### 3.4. Mutation Patterns

The total number of non-synonymous amino acid substitutions in A(H1N1)pdm09, A(H3N2), and Influenza B varied substantially and were distributed throughout all viral proteins. However, only a small subset of substitutions was recurrent (Supplementary Tables S4–S6), defined as having a frequency ≥ 5% within each protein, and was retained for downstream analyses. These recurrent substitutions occurred at frequencies ranging from intermediate to near fixation and most of them across influenza A and B viruses were classified as having low to moderate biological significance (interest levels 0–2) according to FluSurver.

In A(H1N1)pdm09, 430 unique amino acid substitutions were identified compared to the A/Victoria/2570/2019 reference strain. More than half of these substitutions were located in the polymerase complex, particularly PA (28%) and PB2 (25%). Many substitutions were also identified in neuraminidase (NA; 14%) and hemagglutinin (HA; 11%), whereas lower proportions were observed in the remaining proteins. Forty-two substitutions were recurrent (Table S4), predominantly located in HA (*n* = 14), the polymerase complex (*n* = 7), and NA (*n* = 5). Several recurrent substitutions in HA, such as P154S, K159R, Q206E, A203T, and E241A, located in the Ca, Cb antigenic sites, have been previously linked with antigenic drift or host specificity. In contrast, NA substitutions, such as N50D, G382E, and S339L, were annotated as occurring in structural or interaction-related regions and were detected at intermediate frequencies (30–60%), without reaching fixation. Additionally, two substitutions in NS1 (P212S) and NEP (E67G), observed at 60% frequency, have been previously discussed in the context of host–virus fitness.

Regarding A(H3N2), 2977 amino acid substitutions were identified relative to the A/Darwin/9/2021 reference strain. These substitutions were enriched in the polymerase complex, including PA (21%), PB2 (20%), and PB1 (16%), together with HA (11%) and NA (11%), accounting for more than 80% of all substitutions, while fewer substitutions were detected in the other proteins. In total, 80 substitutions were recurrent (Table S5), predominantly identified in HA (*n* = 14), NA (*n* = 15), and PA (*n* = 12). Some HA substitutions, including I156K/M (antigenic site A), S172H and N202D, were detected at variable frequencies and have been associated with antibody recognition sites or antigenic drift. Similarly, NA substitutions, such as S150R, V263I, S331R, and D346G/S, were also detected at variable frequencies and were annotated as potentially related to antigenic drift or neuraminidase inhibitor resistance.

For Influenza B/Victoria, 1053 amino acid substitutions were identified compared to the B/Austria/1359417/2021 reference strain. In contrast to influenza A viruses, these substitutions were more evenly distributed, with NP (16%), HA (16%), PA (16%), PB1 (15%), and NA (14%) having the highest proportions, followed by PB2 (10%). Forty-six of the observed substitutions were recurrent (Table S6), including HA mutations such as D202A (interest level 3), E195K, V102A, and R510K, detected at variable to high frequencies and annotated as antigenic drift, while recurrent NA substitutions, including I459V and V395I, were reported as located in structural or interaction regions. Additional recurrent substitutions with higher interest levels were identified in other viral proteins, including PA (A352T), NP (A28T), and M1 (R105K), reflecting potential roles in host adaptation or viral fitness.

Predicted N-linked glycosylation motifs were examined in HA sequences to identify potential gains or losses associated with amino acid substitutions. Overall, most subtype- and lineage-specific glycosylation profiles were conserved, with only sporadic changes observed. In A(H3N2), the N110S substitution was detected in 5.6% of sequences and was predicted to introduce a new N-linked glycosylation motif within the immunogenic motif D. In contrast, no novel predicted N-linked glycosylation sites were identified among A(H1N1)pdm09 or B/Victoria sequences. Although this A(H3N2) change could potentially alter local antigen exposure, its impact on antibody recognition cannot be inferred from genomic data alone and requires antigenic confirmation.

Recurrent substitutions in the internal proteins of A(H1N1)pdm09, A(H3N2), and Influenza B/Victoria were generally consistent with gradual viral adaptation driven by genetic drift rather than abrupt functional shifts. Although some substitutions were annotated as potentially relevant from a phenotypic or epidemiological perspective, including changes associated with antigenic variation, host adaptation, or antiviral susceptibility, they should be interpreted as sequence-based signals rather than direct evidence of increased virulence, immune escape, or antiviral resistance. No discordant HA/NA subtype combinations were detected using K-FluDB among influenza A samples with sufficient HA and NA recovery [35].

## 4. Discussion

The COVID-19 pandemic caused an unprecedented disruption in the global circulation of seasonal influenza viruses, resulting in a marked reduction in viral activity and genetic diversity between 2020 and 2021. Influenza re-emerged worldwide when interventions were gradually relaxed, providing a unique opportunity to evaluate how influenza evolutionary dynamics re-established after a prolonged period of restricted circulation [10,17,38]. In this study, we present a comprehensive genomic analysis of influenza viruses circulating in Mexico during the first post-pandemic season, contextualized within pre-pandemic and global evolutionary trends.

By generating more than 90% of the influenza virus genomes available from Mexico for the 2022–2023 season, this study shows that post-pandemic influenza circulation in the country was characterized by reduced diversity, consistent with a severe population bottleneck, followed by rapid lineage replacement rather than gradual diversification. Influenza A(H3N2) dominated circulation in Mexico during this period, in agreement with post-pandemic influenza dynamics reported globally and suggesting a synchronized reactivation of influenza patterns reported globally, suggesting a synchronized reactivation of influenza evolutionary dynamics rather than a country-specific trajectory.

Within this framework, the limited circulation of A(H1N1)pdm09 in Mexico was associated with a small number of clades derived from 6B.1A.5a.2a. Globally, this clade and its descendants became dominant after the pandemic [37]. On the other hand, A(H3N2) viruses were dispersed among several subclades derived from 3C.2a1b.2a, indicating greater diversification within globally dominant lineages and likely contributing to their epidemiological predominance during the post-pandemic period. These lineages and their descendants were also dominant in Europe [39]. The delayed re-emergence of the B/Victoria lineage and the nearly exclusive circulation of V1A.3a.2 further suggest expansion from a limited genetic pool, while B/Yamagata viruses remained undetected [40].

Phylogenetic reconstructions based on both HA and NA segments indicate that Mexican influenza sequences were clustered with strains from North and South America, suggesting multiple introductions and strong regional connectivity rather than prolonged local persistence. Such findings are consistent with reports showing that COVID-19 pandemic interventions reshaped the global dispersal of seasonal influenza. In this context, reduced viral diversity and renewed population mobility may facilitate the rapid spread of recently emerged lineages during the early post-pandemic period [41].

At the amino acid level, substitutions were detected across all viral proteins. However, most were associated with low to moderate biological relevance such as antigenic drift, host adaptation, virulence, or antiviral drug interaction, but no canonical markers of enhanced virulence, antiviral resistance, or antigenic escape were found. The substitutions in the polymerase complex, HA, and NA indicate continuous adaptive fine-tuning rather than rapid adaptation. This pattern is consistent with the role of immune and ecological pressures in shaping the evolution of influenza viruses, particularly through antigenic drift, as previously described [42].

Several substitutions detected in this study have also been reported in other countries. For A(H1N1)pdm09, mutations in or near HA antigenic sites, including S154P/T (Ca2) and R240Q, near to the receptor binding region, have been described in sequences from China [43], Italy [44], and Russia [45]. Similarly, mutations I156K, S172H, N202D, and K292R in H3N2, which are located in antigenic regions of HA, have also been reported in sequences from China [43]. The recurrence of these substitutions across countries suggests that the genetic patterns observed in Mexico were part of broader post-pandemic evolutionary trends rather than isolated local events.

The clinical and public health relevance of the observed substitutions should be interpreted with caution. This study did not include antigenic characterization by hemagglutination inhibition or virus neutralization assays, and vaccination status was not available for the sampled individuals. Therefore, the identified mutations should be considered sequence-based signals rather than direct phenotypic or antigenic evidence, and vaccine effectiveness could not be estimated directly. In Mexico, seasonal influenza vaccination is recommended for children younger than 5 years of age, adults 60 years and older, pregnant women, health workers, and individuals with risk factors [46]. However, previous reports indicate that vaccine coverage can remain incomplete even within target populations, with estimates varying by year and public health institution [47].

Nevertheless, the predominance of lineages similar to those reported in the United States and Europe provides useful context for interpreting the potential public health relevance of the observed genetic patterns. During comparable post-pandemic seasons, vaccine effectiveness estimates varied by subtype and region. Generally moderate protection was observed in the United States, with overall effectiveness reported to be around 30% [48]. However, during the same period, European estimates were 11% against A(H1N1)pdm09, 20% against A(H3N2), and 56% against influenza B [49]. Future studies integrating genomic surveillance, antigenic assays, and vaccination data will be necessary to assess the impact of these substitutions on antigenicity, vaccine match, antibody recognition, and vaccine effectiveness.

A limitation of this study is that genomic sampling was not geographically uniform across Mexico. Although sequencing output closely followed the temporal distribution of confirmed influenza cases, regional sampling did not fully mirror reported influenza activity. W, NE, and CS were relatively proportional to their reported case burden, whereas SE was over-represented and CN was under-represented. This imbalance may limit the detection of low-frequency lineages, localized introductions, or short transmission chains in under-sampled regions. Therefore, lineage turnover and regional connectivity patterns should be interpreted as signals from the available genomic surveillance dataset rather than as a complete reconstruction of influenza circulation across all regions of Mexico. In addition, regional-level analyses could not be consistently performed because several regions had few genomes across multiple months; thus, the evolutionary and epidemiological patterns presented here largely reflect national-level dynamics.

Another technical limitation was the lower recovery of the PB1 segment. This may reflect its large size, RNA quality, viral load, amplification efficiency, or primer–template mismatches in the adapted sequencing workflow. Although this could limit the detection of reassortment involving internal segments, it does not affect the HA/NA-based K-FluDB screening, as HA and NA were recovered from most samples. Thus, the absence of reassortment signals refers specifically to the lack of discordant HA/NA subtype combinations.

Taken together, our results show that the post-pandemic re-emergence of influenza viruses in Mexico was marked by reduced diversity, fast lineage replacement, and strong regional connectivity, mirroring broader global patterns. As influenza circulation continues to normalize, these observations underscore the importance of sustained genomic surveillance to identify early indicators of antigenic drift, lineage growth, or alterations in virus population structure.

## Data Availability

The data generated in this work can be retrieved in the EpiFlu GISAID database using the accession number EPI_SET_260526vu. Additional data used in the analysis can be retrieved with the accession numbers EPI_SET_260319uq and EPI_SET_260204tx.

## Supplementary Materials

The following supporting information can be downloaded at: https://www.mdpi.com/article/10.3390/v18060609/s1; Figure S1. Geographic regionalization used for the assessment of influenza genomic sampling bias in Mexico; Figure S2. Temporal dynamics of HA influenza virus clades circulating in Mexico (2018–2023). Monthly relative frequencies of HA clades for (A) influenza A(H1N1)pdm09, (B) influenza A(H3N2), and (C) influenza B/Victoria. Relative frequencies of influenza B/Yamagata clades were also evaluated but are not shown due to the disappearance of this lineage after the COVID-19 pandemic. Only months with at least 17 sequences were included to ensure reliable estimation of relative clade frequencies; Figure S3. Maximum likelihood phylogenetic tree for NA Influenza A subtype N1. Tips of the tree are colored by sampling location, and the heatmap alongside the tips show the NA clade assignment of each sequence. The vaccine strain contemporary to the Mexican sequences reported in this study is marked in black; Figure S4. Maximum likelihood phylogenetic tree for NA Influenza A subtype N2. Tips of the tree are colored by sampling location, and the heatmap alongside the tips show the NA clade assignment of each sequence. The vaccine strain contemporary to the Mexican sequences reported in this study is marked in black; Figure S5. Maximum likelihood phylogenetic tree for NA Influenza B subtype Victoria. Tips of the tree are colored by sampling location, and the heatmap alongside the tips show the NA clade assignment of each sequence. The vaccine strain contemporary to the Mexican sequences reported in this study is marked in black; Table S1. GISAID isolate identifiers and GenBank accession numbers for each segment of influenza virus genomes from Mexico collected between 1 June 2022, and 30 June 2023; Table S2. Summary of genomic segment recovery across influenza virus subtypes from Mexico collected between 1 June 2022, and 30 June 2023; Table S3. Regional comparison between sequenced influenza genomes and laboratory-confirmed influenza cases in Mexico during June 2022–June 2023; Table S4. Recurrent amino acid substitutions in influenza A(H1N1)pdm09 viruses circulating in Mexico; Table S5. Recurrent amino acid substitutions in influenza A(H3N2) viruses circulating in Mexico; Table S6. Recurrent amino acid substitutions in influenza B/Victoria viruses circulating in Mexico. Table S7. FluNexus-based antigenic prediction of A/H3N2 HA sequences relative to the A/Darwin/9/2021 vaccine-strain reference. Table S8. FluNexus-based antigenic prediction of A/H1N1pdm09 HA1 sequences relative to the A/Victoria/2570/2019 vaccine-strain reference.

## Abbreviations

The following abbreviations are used in this manuscript:

GIHSN	Global Influenza Hospital Surveillance Network
GISAID	Global Initiative on Sharing All Influenza Data
GISRS	Global Influenza Surveillance and Response System
HA	Hemagglutinin
IMSS	Instituto Mexicano del Seguro Social
InDRE	Instituto de Diagnóstico y Referencia Epidemiológicos
INER	Instituto Nacional de Enfermedades Respiratorias
M1	Matrix protein 1
M2	Matrix protein 2
NA	Neuraminidase
NP	Nucleoprotein
NS1	Non-structural protein 1
NS2/NEP	Nuclear export protein/Non-structural protein 2
PA	Polymerase acidic protein
PB1	Polymerase basic protein 1
PB2	Polymerase basic protein 2
RNA	Ribonucleic acid
WHO	World Health Organization
